# A retrospective evaluation of the Brain and Body Fitness Studio service on functional capacity and quality of life in people with neurological disorders

**DOI:** 10.3389/fneur.2022.1006221

**Published:** 2023-01-20

**Authors:** Joyce S. Ramos, Ranjay Chakraborty, Lance C. Dalleck, Kristina Sarunic, Jyoti Khadka, Tayla Haslam, Olivia Nassaris

**Affiliations:** ^1^Clinical Exercise Physiology, College of Nursing and Health Sciences, SHAPE Research Centre, Caring Futures Institute, Flinders University, Adelaide, SA, Australia; ^2^Recreation, Exercise and Sport, Western Colorado University, Gunnison, CO, United States; ^3^Health and Social Care Economics Group, College of Nursing and Health Sciences, Caring Futures Institute, Flinders University, Adelaide, SA, Australia; ^4^Brain and Body Fitness Studio, The Hospital Research Foundation Parkinson's, Adelaide, SA, Australia

**Keywords:** functional capacity, fitness, disease progression, neurological disorder, mobility, Parkinson's disease quality of life, exercise

## Abstract

**Background:**

People with neurological disorders (ND) are less physically active than the general population due to physical, sensory, and/or cognitive impairments. These individuals often feel intimidated to join mainstream health and wellness centers due to lack of specialized support for people with ND. The Brain and Body Fitness Studio (BBFS) is one of the first Accredited Exercise Physiologist-led interprofessional services in Adelaide South Australia to provide individualized evidence-based multimodal exercise prescription and social support for this population. This comprehensive retrospective study evaluated the impact of BBFS on functional capacity (FC) determined as the 6-min walk distance (6 MWD) achieved during a 6-min walk test (6 MWT), of its members with ND.

**Methods:**

Sixty-two BBFS members (age, 66 ± 10 years; 60% male) with ND (85% Parkinson's Disease; average time since diagnosis, 4 years [IQR, 2 to 12 years]) and complete pre- and post-6-month clinical assessment of the primary outcome of the study, the 6 MWD, were included in this retrospective analysis. A series of sub-analyses were also performed to investigate the effects of adherence to the recommended prescription of at least twice a week in the program (≥80 vs. < 80% adherence), and disease stage (time since diagnosis; ≥6 vs. < 6 years) on FC.

**Results:**

Although there was no statistically significant change in 6 MWD from pre- to post-6-month BBFS program (+15 ± 90 m, *p* = 0.19), a clinically meaningful improvement of >14 m was evident. Improvement in 6 MWD was significantly greater in members who attended at least 80% of the recommended visits (≥80% visits, +37 ± 58 m; ≤ 80% visits,−1 ± 105 m, *p* = 0.046). We also found a 6 MWD improvement from pre- to post-6 months in those in the early years of their ND (< 6 years since diagnosis, +39 ± 76 m), but not in those in the later years of their ND (≥6 years since diagnosis, −36 ± 123 m, between group difference, *p* = 0.029).

**Conclusion:**

A clinically meaningful 6 MWD improvement may be elicited by services provided by BBFS in people with ND. Overall, the benefits appear to be more evident in members who attended the BBFS for at least 80% of the recommended visits and those who were in the early stage of their ND diagnosis.

## 1. Introduction

Neurological disorders (ND) are the most common cause of disability worldwide, imposing a significant economic burden on the health care system (1). People with ND usually suffer from physical, sensory, and/or cognitive impairments, and are consequently less physically active than the general population (2). Hence, people diagnosed with ND are at high risk of secondary conditions such as hypertension, heart disease, type 2 diabetes, and obesity (3). The interrelationship of cognitive deficits and motor symptoms present in this cohort also result in a struggle to adapt to a changing environment and multiple tasks, which are often required for daily tasks such as gait and balance (4). Thus, as the disease progresses, people with ND become highly vulnerable to falls and fall-related injuries (5). There is therefore a need for well-designed exercise programs for improving functional capacity (FC), falls risk, and health-related quality of life (QOL) in this population.

The 6-min walk distance (6 MWD) achieved during a 6-min walk test (6 MWT) is a widely used method to assess treatment efficacy and disease progression in people with ND (6–8). This is because walking capacity is an important determinant of FC to independently perform activities of daily living (6, 7), which inevitably is expected to translate to better QOL (9). In addition, this test has also been demonstrated to serve as screening tool to detect falls risk in this population (10). However, the impact of a holistic individualized evidence-based multimodal exercise program on this important outcome measure in people with ND has yet to be investigated.

Low engagement of this cohort to regular exercise programs has been reported to be due to multiple barriers ranging from personal to environmental barrier including low levels of self-efficacy, lack of social support, and restricted access to beneficial physical activity/exercise programs (11). Importantly, those newly diagnosed with ND are often either neglected by the health system until later in the disease stage or feel intimidated and diffident to join mainstream wellness centers that do not often offer specialized support for people with neurological conditions (12). The Brain and Body Fitness Studio (BBFS) is an interprofessional center-based clinic in Adelaide South Australia that provides services that focus on body and brain health through provision of individualized evidence-based exercise prescription and social support. It includes members who have been diagnosed with ND, predominantly Parkinson's disease. Anecdotal evidence suggests that participation in exercise services provided by BBFS significantly improves FC and overall health-related QOL in people with neurological conditions.

This study therefore aimed to conduct a comprehensive retrospective evaluation of the impact of BBFS on FC, falls risk, and health-related QOL of its members with ND who underwent 6-months of an evidence-based individualized exercise program at the center-based clinic. Specifically, the primary outcome of this study was the change in 6 MWD, as assessed by the 6 MWT. Secondary outcomes were other FC, fall risk, disease progression, and overall QOL.

## 2. Methodology

BBFS members diagnosed with a ND (Table 1) were included in this retrospective analysis. The BBFS offers paid private membership or national disability insurance scheme (NDIS) supported membership to the patients. Only those with complete pre- and post-6-month data of the primary outcome (6 MWD) of the study were included. Several sub-analyses were performed to account for: adherence to the program, changes in the dosage or type of medications affecting dopamine levels from pre- to post-intervention (Levodopa/Benserazide; Levodopa/Carbidopa; Dopamine Agonist; decarboxylase inhibitors), and the time since diagnosis of a ND. We first compared the changes in clinical outcome measures in those who attended at least 80% vs. < 80% of the recommended BBFS visits of twice per week (Table 2). A subsequent sub-analysis was then performed which excluded participants who had changed their dopamine-related medications following the intervention timepoints (Table 3). Finally, we compared changes in all clinical outcome measures following all intervention timepoints between those diagnosed with a ND for at least 6 years relative to those who were only diagnosed with a ND for < 6 years. This study was approved by the Human Research Ethics Committee at Flinders University (project no. 4528). Written informed consent were obtained from all participants before inclusion of their data in this retrospective analysis.

**Table 1:** Demographics and clinical characteristics of participants at baseline.

**6-month data**	**ALL (*n* = 62)**	**≥80% Adherence (*n* = 26)**	** < 80% Adherence (*n* = 36)**
Age, years (mean ± SD)	66 ± 10	67 ± 10	66 ± 10
Male, sex (%)	60	65	56
Parkinson's Disease (%)	85	96	78
Time since diagnosis, years (median [IQR])	4 (2 to 12)	4 (2 to 10)	4 (3 to 14)
Adherence (%)	76	104	56

Data are presented as mean ± SD or median (IQR).

**Table 2:** Summary of 6-month data.

	**ALL (*****n*** = **62)**				≥**80% Adherence (*****n*** = **26)**			<**80% Adherence (*****n*** = **36)**			**≥80 vs.** **< 80%** **Adherence**
**Outcome measures**	**Baseline**	**6-months**	Δ	**Within-group** ***p*****-value**	**Baseline**	**6-months**	Δ	**Baseline**	**6-months**	Δ	**Between-group**, ***p*****-value**
**Functional capacity and fall risk**											
**Exercise capacity and muscular strength**											
6 MWD (m)	417 ± 125	432 ± 126	15 ± 90	0.19	431 ± 127	468 ± 129	37 ± 58	408 ± 124	407 ± 119	−1 ± 105	0.046
30 s STS (reps)	11 (9 to 14)	13 (10 to 15)	2 (−1 to 3)	< 0.01	11 (9 to 14)	13 (11 to 15)	−1 (4 to 5)	11 (9 to 14)	12 (10 to 14)	1 (−1 to 3)	0.136
TUG R side (s)	9 (7 to 10)	8 (7 to 10)	−1 (−1 to 0)	0.05	8 (7 to 10)	7 (6 to 9)	−1 (−1 to 0)	9 (7 to 9)	9 (7 to 10)	0 (−1 to 1)	0.282
TUG L side (s)	8 (7 to 10)	8 (7 to 10)	0 (−1 to 1)	0.19	8 (6 to 9)	8 (6 to 9)	0 (−1 to 1)	8 (8 to 10)	9 (7 to 10)	0 (−1 to 1)	0.507
30 s Bicep curl R arm (reps)	17 (14 to 18)	18 (15 to 20)	1 (−1 to 3)	< 0.01	17 ± 4	19 ± 5	2 ± 3	16 ± 4	17 ± 4	1 ± 3	0.079
30 s Bicep curl L arm (reps)	16 (13 to 18)	17 (15 to 20)	1 (0 to 3)	< 0.001	16 (13 to 18)	17 (16 to 21)	2 (0 to 4)	15 ± 4	16 ± 4	1 ± 3	0.084
**Flexibility**											
Hamstring R side (cm)	−15 ± 14	−12 ± 14	3 ± 11	0.02	−15 ± 16	−11 ± 15	4 ± 14	−15 ± 13	−13 ± 13	3 ± 9	0.602
Hamstring L side (cm)	−15 ± 15	−12 ± 14	3 ± 11	0.02	−16 ± 16	−11 ± 15	5 ± 14	−15 ± 14	−13 ± 14	3 ± 8	0.466
Shoulder R side (cm)	−15 (−29 to −7)	−14 (−27 to −4)	0 (-2 to 3)	0.016	−19 ± 14	−16 ± 14	2 ± 7	−13 (−26 to −5)	−13 (−18 to −3)	0 (−3 to 2)	0.405
Shoulder L side (cm)	−23 ± 13	−22 ± 13	1 ± 6	0.20	−23 ± 13	−21 ± 11	2 ± 6	−23 ± 13	−23 ± 14	0 ± 7	0.150
**Postural stability**											
SLS R eyes open (s)	13 (4 to 15)	14 (7 to 15)	0 (0 to 3)	0.05	15 (6 to 5)	15 (10 to 15)	0 (0 to 2)	9 (4 to 15)	12 (5 to 15)	0 (−1 to 3)	0.109
SLS L Eyes Open (s)	12 (4 to 15)	13 (4 to 15)	0 (−1 to 1)	0.82	15 (8 to 15)	15 (8 to 15)	0 (0 to 1)	9 (3 to 15)	8 (4 to 15)	0 (−2 to 1)	0.522
Tandem R (s)	15 (9 to 15)	15 (14 to 15)	0 (0 to 2)	0.12	15 (11 to 15)	15 (15 to 15)	0 (0 to 2)	15 (8 to 15)	15 (11 to 15)	0 (0 to 1)	0.727
Tandem L (s)	15 (15 to 15)	15 (15 to 15)	0 (0 to 0)	0.44	15 (15 to 15)	15 (15 to 15)	0 (0 to 0)	15 (13 to 15)	15 (11 to 15)	0 (0 to 0)	0.371
**Health-related questionnaires**											
**Functional capacity and fall risk**											
Modified Frop-Com	3 ± 5	2 ± 2	−1 ± 5	0.39	4 ± 7	3 ± 2	−1 ± 7	2 ± 2	2 ± 2	0 ± 2	0.896
NFOG-Q	9 ± 4	9 ± 5	1 ± 6	0.49	9 ±4	9 ± 4	0 ± 7	8 ± 4	10 ± 6	2 ± 6	0.195
FESI	32 ± 12	32 ± 13	1 ± 12	0.76	33 ± 9	30 ± 10	−3 ± 14	31 ± 14	34 ± 14	4 ± 10	0.180
**Quality of life**											
PDQ-39	33 (16 to 40)	32 (21 to 40)	3 (−3 to 10)	0.16	32 ± 12	29 ± 15	−2 ± 13	29 ± 18	36 ± 18	7 ± 17	0.159
SF12–CS-12	38 ± 8	40 ± 10	1 ± 10	0.29	38 ± 8	38 ± 8	0 ± 9	39 ± 8	41 ± 10	2 ± 10	0.346
SF12– MCS-12	47 ± 10	47 ± 11	0 ± 10	0.99	46 ± 10	46 ± 11	1 ± 8	49 ± 10	47 ± 11	−1 ± 12	0.819
EQ-5D-3L Health Index	0.67 ± 0.20	0.64 ± 0.22	−0.03 ± 0.21	0.342	0.66 ± 0.16	0.62 ± 0.21	−0.04 ± 0.19	0.67 ± 0.23	0.66 ± 0.23	−0.01 ± 0.23	0.461
EQ-5D-3L VAS	68.49 ± 16.48	72.14 ± 13.74	3.65 ± 15.11	0.416	69.41 ± 11.46	74.43 ± 12.63	5.02 ± 12.04	67.34 ± 13.56	70.13 ± 15.37	2.79 ± 14.47	0.372
EXSE	20 (15 to 24)	20 (15 to 24)	−1 (−4 to 2)	0.48	21 ± 7	20 ± 7	−1 ± 8	21 ± 8	20 ± 7	0 ± 8	0.716

Data are presented as mean ± SD or median (IQR) and mean change ± median change (IQR).

**Table 3:** Summary of results excluding those with a change in dopamine-related medication from pre- to post-6-month intervention.

	**No dopamine-related medication changes from pre- to post-6-month intervention (*****n*** = **49)**				≥**80% Adherence** + **no dopamine-related medication changes from pre- to post-6-month intervention (*****n*** = **23)**			<**80% Adherence** + **no dopamine-related medication changes from pre- to post-6-month intervention (*****n*** = **26)**			**Between-group**
**Outcome measures**	**Baseline**	**6-months**	Δ	**Within-group** ***p*****-value**	**Baseline**	**6-months**	Δ	**Baseline**	**6-months**	Δ	***p*** **-value**
**Functional capacity and fall risk**											
**Exercise capacity and muscular strength**											
6 MWD (m)	426 ± 122	436 ± 120	10 ± 89	0.417	444 ± 115	482 ± 109	39 ± 60	410 ± 128	395 ± 115	−15 ± 102	0.006
30 s STS (reps)	11 ± 4	13 ± 4	1 ± 3	0.006	12 ± 5	14 ± 4	2 ± 3	11 ± 4	12 ± 4	1 ± 3	0.036
TUG R side (s)	8 (7 to 9)	8 (6 to 9)	−1 (−1 to 0)	0.023	9 ± 3	8 ± 2	−1 ± 2	9 ± 4	9 ± 3	0 ± 2	0.005
TUG L side (s)	8 (6 to 9)	8 (6 to 9)	0 (−1 to 1)	0.162	8 (6 to 9)	8 (6 to 9)	−1 (−1 to 0)	9 (8 to 9)	9 (8 to 11)	0 (−1 to 1)	0.086
30 s Bicep curl R arm (reps)	17 ± 4	18 ± 5	1 ± 3	0.003	17 ± 4	20 ± 5	3 ± 3	16 ± 3	16 ± 4	0 ± 2	0.007
30 s Bicep curl L arm (reps)	16 ± 4	17 ± 5	2 ± 3	< 0.001	16 ± 4	19 ± 5	3 ± 3	15 ± 4	16 ± 4	0 ± 2	0.007
**Flexibility**											
Hamstring R side (cm)	−17 ± 14	−14 ± 14	3 ± 11	0.08	−15 ± 16	−12 ± 16	3 ± 14	−18 ± 12	−16 ± 13	2 ± 8	0.582
Hamstring L side (cm)	−17 ± 15	−13 ± 15	4 ± 12	0.031	−14 (−25 to−4)	−12 (−21 to 0)	5 (1 to 12)	−14 (−30 to −10)	−13 (−25 to −9)	2 (−2 to 8)	0.212
Shoulder R side (cm)	−19 ± 15	−17 ± 15	1 ± 8	0.166	−20 (−31 to 8)	−16 (−27 to −4)	2 (−1 to 4)	−15 (−27 to −9)	−16 (−29 to −8)	−1 (−3 to 0)	0.058
Shoulder L side (cm)	−24 ± 14	−23 ± 13	0 ± 6	0.630	−23 ± 14	−20 ± 12	2 ± 6	−25 ± 14	−27 ± 13	−2 ± 5	0.001
**Postural stability**											
SLS R eyes open (s)	12 (5 to 15)	14 (6 to 15)	0 (0 to 3)	0.064	14 (5 to 15)	15 (10 to 15)	0 (0 to 3)	9 (4 to 15)	12 (5 to 15)	0 (−1 to 2)	0.090
SLS L eyes open (s)	12 (3 to 15)	13 (5 to 15)	0 (−1 to 1)	0.550	15 (9 to 15)	15 (9 to 15)	0 (−1 to 1)	6 (3 to 15)	8 (4 to 15)	1 (0 to 2)	0.908
Tandem R (s)	15 (8 to 15)	15 (15 to 15)	0 (0 to 5)	0.069	15 (11 to 15)	15 (15 to 15)	0 (0 to 1)	12 (8 to 15)	15 (9 to 15)	0 (0 to 5)	0.815
Tandem L (s)	15 (9 to 15)	15 (15 to 15)	0 (0 to 3)	0.093	15 (15 to 15)	15 (15 to 15)	0 (0 to 0)	15 (8 to 15)	15 (11 to 15)	0 (0 to 2)	0.405
**Health related questionnaires**											
**Functional capacity and fall risk**											
Modified Frop-Com	2 (0 to 4)	2 (0 to 4)	0 (-1 to 1)	0.89	3 (0 to 5)	3 (0 to 4)	0 (-1 to 2)	1 (0 to 3)	1 (0 to 3)	0 (-1 to 1)	0.608
NFOG-Q	9 ± 4	10 ± 5	1 ± 6	0.327	9 ± 4	9 ± 5	1 ± 8	9 ± 4	11 ± 5	3 ± 6	0.180
FESI	32 ± 12	32 ± 12	0 ± 12	0.864	33 ± 9	29 ± 9	−4 ± 13	31 ± 15	36 ± 14	5 ± 9	0.020
**Quality of life**											
PDQ-39	30 ± 16	33 ± 17	3 ± 15	0.404	31 ± 12	27 ± 13	−4 ± 13	29 ± 18	38 ± 19	8 ± 15	0.051
SF12P	38 ± 9	39 ± 9	1 ± 10	0.629	38 ± 8	39 ± 8	1 ± 9	38 ± 9	39 ± 10	2 ± 10	0.942
SF12M	47 ± 10	45 ± 11	−1 ± 10	0.363	45 ± 10	46 ± 11	0 ± 7	48 ± 10	45 ± 11	−3 ± 12	0.494
EQ-5D-3L Health Index	0.64 ± 0.17	0.65 ± 0.21	0.01 ± 0.19	0.361	0.65 ± 0.14	0.68 ± 0.21	0.03 ± 0.18	0.63 ± 0.22	0.62 ± 0.19	−0.01 ± 0.21	0.416
EQ-5D-3L VAS	71.64 ± 14.11	73.18 ± 11.66	1.54 ± 12.89	0.574	69.64 ± 12.42	74.11 ± 14.73	4.47 ± 13.58	73.34 ± 14.82	72.41 ± 13.31	−0.93 ± 14.07	0.428
EXSE	21 ± 8	20 ± 7	−1 ± 9	0.631	21 ± 7	19 ± 7	−2 ± 8	21 ± 9	21 ± 7	0 ± 9	0.329

Data are presented as mean ± SD or median (IQR) and mean change ± median change (IQR).

### 2.1. Clinical outcome measures

Objective and subjective clinical outcome assessments were conducted during the participants' “ON” state, at approximately the same time of the day and sequence at pre- and post-exercise program. The objective measures were administered in the following order at each timepoint: 30 s sit-to-stand (30 s STS) test, 30 s bicep curl test, hamstring flexibility, shoulder flexibility, 3 m timed up and go test (TUG), 6 MWT, and questionnaires (subjective measures of functional capacity and fall risk; QOL, and self-efficacy for exercise).

#### 2.1.1. 6-min walk distance

The primary outcome measure was the total distance covered during the 6 MWT, which is a measure of functional capacity (6) and fall risk (10). This assessment required participants to walk up and down a 15 m marked circuit allocated at the BBFS facility. The participants were instructed to walk as far and as fast as possible for 6 min, and to slow down or rest at any point during the test if required. A tester was available to supervise the performance of turns at the endpoints of the 15 m lane. The total distance (m) covered during the 6 MWT (6 MWD) was recorded.

#### 2.1.2. Other objective measures of functional capacity and fall risk

Data from the following objective assessments of functional capacity and falls risk at each study time point were collated and analyzed: 30 s sit-to-stand (30 s STS) test, 30 s bicep curl test, hamstring flexibility (chair sit and reach test), shoulder flexibility test (back scratch); 3 m timed up and go test, tandem balance test, and single leg stance test.

##### 2.1.2.1. 30 s Sit-to-Stand test

The 30 s STS test required participants to repetitively stand up and sit down from a standard chair positioned against the wall, as fast and as safely as possible within 30 s to estimate lower body muscle strength. The successful completion of one cycle of standing up and sitting down from a chair was counted as one repetition. The number of repetitions completed during the 30 s STS test was recorded (13).

##### 2.1.2.2. 30 s Bicep curl test

The 30 s bicep curl test was used to assess upper body strength. This test was performed in a seated position, with the back straight and feet flat on the floor. Before the start of the test, a dumbbell (2 kg for females or 3 kg for males) was held in a neutral handshake grip position, with the arm hanging down the side of the participant's body. The test required the participant to flex at the elbow while supinating the forearm and return to the starting position. This was repeated as fast as possible in 30 s, whilst ensuring that that the elbow is positioned against the trunk during the curls. The number of repetitions completed in 30 s was recorded for each arm (left and right) (13).

##### 2.1.2.3. Hamstring flexibility

The chair sit and reach test was conducted to determine hamstring flexibility, a functional measure of the left and right hamstring region (14). The participants sat on a chair placed against the wall, with their inguinal fold positioned parallel to the edge of the chair. At the start of the test, one leg was bent with the foot flat on floor, whilst the opposite leg extended as straight as possible toward the front of the hip with the heel in contact with the floor and the foot at a 90-degree dorsiflexed position. The participants were then instructed to slowly reach their toes with both hands as far as possible, holding the maximum position momentarily. Both hands were maintained in a parallel position, not allowing one hand to reach further than the other, with the fingertips overlapped and in contact with the ruler used to measure the distance reached. All participants completed three trials for each leg (left and right leg), with a minimum of 5 s rest between trials. The best trial from each leg tested was recorded. A positive number was recorded if the participants reached past their toes. If the participants failed to reach their toes, a negative number was recorded. The distance reached to nearest 0.1 of a cm was recorded.

##### 2.1.2.4. Shoulder flexibility

The back scratch test was used to assess shoulder flexibility, as a measure of functional capacity of the left and right shoulder joint (15). The starting position of this test required participants to place one hand on the lower back and the other hand behind the neck. The participants were then instructed to move their hands together, with the aim of placing the long finger of each hand as close as possible. The gap between the fingertips of each hand was measured to the nearest cm. All participants completed three trials for each shoulder (left and right shoulder) located in opposite initial positions as described above. The best trial from each shoulder tested was recorded. Positive numbers were recorded if the long fingers between hands met, whilst negative numbers if the fingers did not overlap.

##### 2.1.2.5. Tandem and single leg stance test

The 15 s tandem and SLS test were conducted to determine postural stability or balance, as an assessment of fall risk (14). The tandem test required participants to maintain an upright balanced position for 30 s whilst the heel of one foot was positioned in front and touching the toes of the other foot. The participant was then instructed to stand upright on one foot with eyes open and without hand support for as long as possible up to 15 s. The test was terminated if the participant reached the 15 s mark or when the participant touched their unsupported foot to the floor or standing leg or touched something to regain postural stability. If the participant was unable to maintain balance for 15 s, a second attempt was provided. The test was conducted on both legs (right and left legs). The highest time to the nearest 0.1 of a second was recorded if the participant was unable to hold the position for 15 s.

#### 2.1.3. Subjective measures of functional capacity and fall risk

Subjective assessments of functional capacity and fall risk at each study time point were also performed only for BBFS members diagnosed with Parkinson's Disease using Falls risk for older people in the community screen (FROP-Com) (16); New Freezing of Gait (NFOG-Q) (17–19); Falls efficacy scale - International (FESI) (20, 21).

#### 2.1.4. Quality of life and Self-efficacy for exercise

The impact of services provided by BBFS on QOL and self-efficacy for exercise were assessed using 12-item short form survey (SF-12) (22, 23), EQ-5D-3L (24–26), and Exercise self-efficacy questionnaire (ESS) (27), From the SF-12 questionnaire, the mental component summary score (MCS-12) and the physical component summary score (PCS-12) were derived using an online score calculator (28), as described previously (29). For the ED-5D-3L questionnaire, the health state index score was calculated from individual health profiles using the time trade-off derived EQ-5D weights for Australia (30). In addition, patients also rated their overall perceived health on the EQ VAS or visual analog scale at each visit (26). Finally, a short 39-item Parkinson's Disease questionnaire (PDQ-39) was only administered to BBFS members diagnosed with Parkinson's disease to assesses Parkinson's disease specific health related quality over the past month (31, 32).

### 2.2. Individualized evidence-based exercise program

The prescription and delivery of individualized evidence-based exercise programs at BBFS was conducted by an AEP in collaboration with other Allied Health Professionals (Occupational Therapist and Physiotherapists), nurse practitioner, and the client's neurologists or GPs when required. Fundamentally, the exercise programs were developed according to the Parkinson's Foundation (United States) exercise guidelines, which encompasses the American College of Sports Medicine general exercise recommendations for people with ND (33), and additional evidence-based information specific to Parkinson's Disease. The exercise programs generally targeted the main fitness components [aerobic, resistance, neuromotor (balance, agility, and multi-tasking)], and flexibility, according to the needs, goals, and disease-related considerations of the clients which were identified during the initial assessment. Alternative evidence-based exercise modes such as boxing (34), dancing (35), forced rate cycling (36), and yoga (37) were also implemented in individual or group class sessions, particularly in clients diagnosed with Parkinson's Disease. The exercise programs typically included 8–12 exercises per session, depending on the clients' cognitive capacity to transition through their program within the allocated 1-h session. All AEPs involved in the prescription and delivery of exercise programs were required to regularly complete professional development programs or courses such as Parkinson's Disease (PD) warrior course (38) to keep up to date with practical and research evidence, to uphold evidence-based practice.

A comprehensive initial assessment was conducted by an AEP with all incoming members of the BBFS clinic to determine their needs, goals, and disease-related considerations. If required, this was subsequently discussed with the inter-professional healthcare team to assist in the development of an individualized evidence-based exercise program. The first session of the exercise program served to educate the clients on the safety aspects of the studio equipment, set-up and storage of equipment, and correct technique. This session was also used to determine the appropriate volume of resistance (sets/repetitions/load) and cardiorespiratory fitness training (intensity/duration) and adjustment of the exercise program if the intended prescription was found to be unsuitable or unsafe, based on clinical reasoning of the supervising AEP. The framework behind the resistance training progression was that once the client was able to comfortably complete 15 repetitions of an exercise with the correct technique, the load (generally machine weight or dumbbell) was able to be progressed. The intensity of each exercise session was monitored using the rate of perceived exertion on the Borg scale (39). The exercise programs were reviewed every 12 weeks, which involved a one-on-one discussion on the clients' progress, goals, attendance, and any updates related to their medical status.

### 2.3. Statistical analyses

Data were analyzed using the SPSS version 27 package (IBM, New York, NY, USA) and SigmaPlot version 13 (Systat Software Inc). The assumption of data normality was tested using the Shapiro-Wilk test to determine the appropriateness of parametric tests. When the assumption of normality was violated, the data were log transformed. A non-parametric test equivalent was used if the normality assumption was still violated after log transformation. A paired *t*-test or Wilcoxon test determined whether there is a significant change in continuous outcome variables from pre-to post-6-month intervention (Tables 2, 3). Repeated measures ANCOVA or a non-parametric equivalent test (Quade's ANCOVA) were used to determine if there is a “group-by-time” interaction or a difference in change in continuous variables from pre- to post-intervention between adherence (≥80 vs. < 80% Adherence) and disease stage groups (time since diagnosis; ≥6 vs. < 6 years) (40). The significance level was set at *p* < 0.05. Continuous variables were reported as mean ± standard deviation (SD) or median (interquartile range [IQR]).

## 3. Results

**Figure 1:**
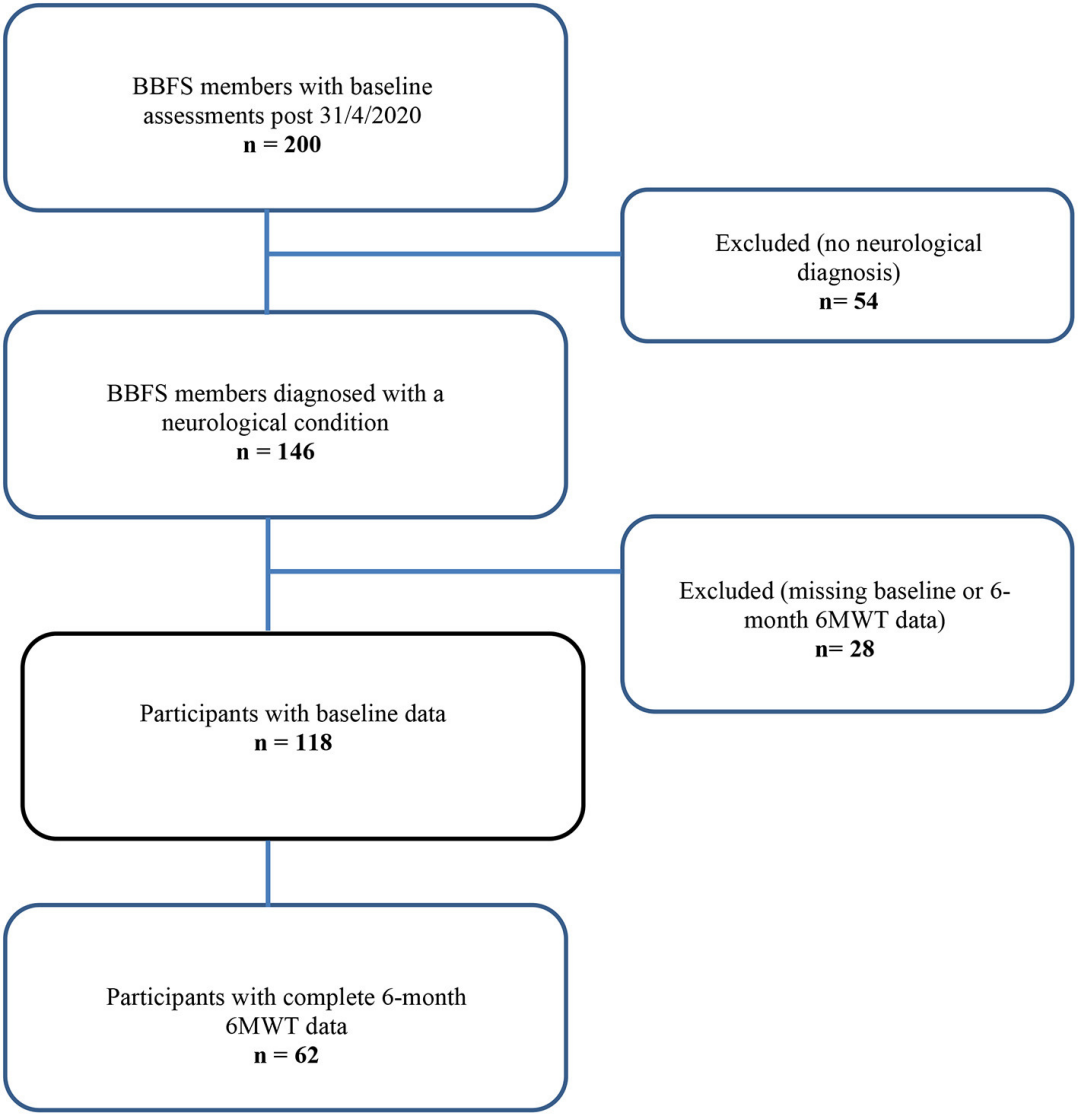
A flow chart of our retrospective study design.

Sixty-two BBFS members (age, 66 ± 10 years; 60% males) diagnosed with a ND (85% Parkinson's Disease, *n* = 53; progressive supranuclear palsy, *n* = 2; motor neurone disease, *n* = 1; essential tremor, *n* = 1; younger onset dementia, *n* = 1; traumatic brain injury, *n* = 1; multiple sclerosis, *n* = 1; corticobasal syndrome, *n* = 1; vestibular migraines, *n* = 1; time since diagnosis, 4 years [IQR, 2 to 12 years]) had complete 6-month data for the primary outcome of the study (Figure 1). Table 1 presents the participants' characteristics at baseline. Only 26 (42%) of the 62 BBFS members with complete 6-month data adhered to at least 80% of the recommended BBFS visits. There were no reported physical injuries that were directly related to the prescribed exercise program.

### 3.1. Six-minute walk distance

**Figure 2:**
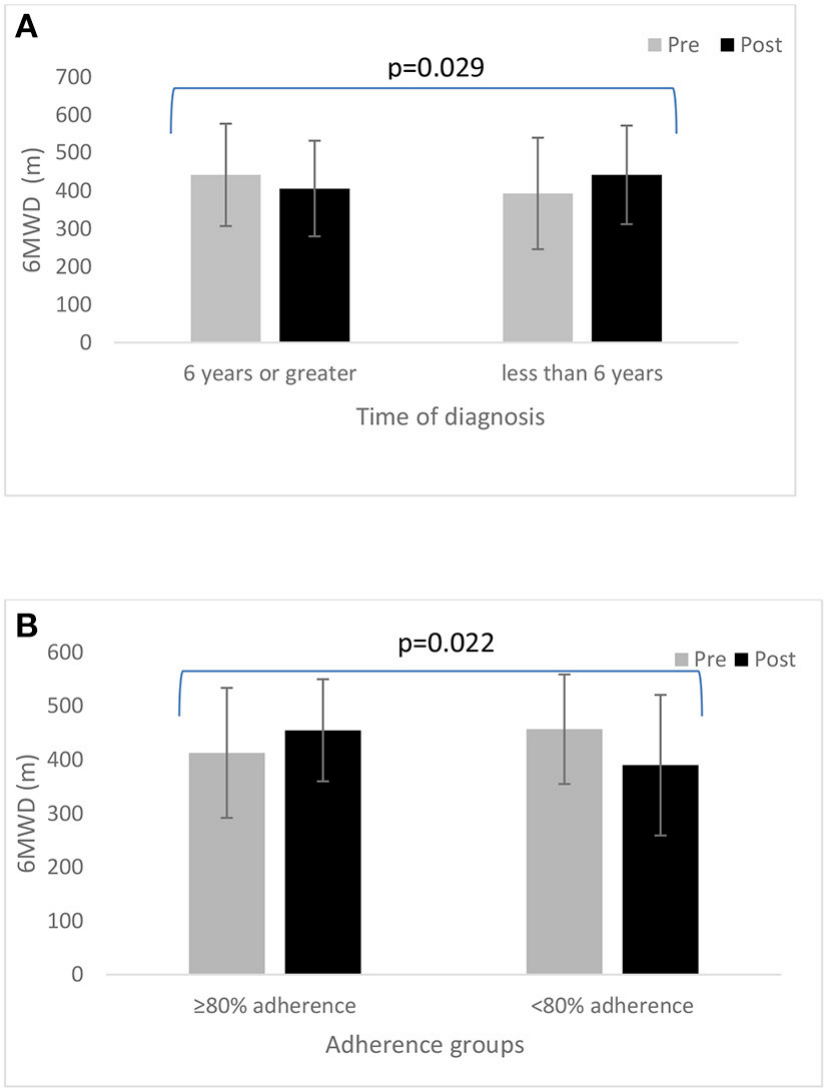
**(A)** Change in 6 MWD from pre- to post-6-month BBFS program between those diagnosed with a neurological disorder for at least 6 years vs. < 6 years. **(B)** A comparison of 6MWD from pre- to post-6-month BBFS program between adherence groups (≥80 vs. < 80%) in those diagnosed with a neurological disorder for at least 6 years.

In all participants, we found no statistically significant change in 6 MWD from pre- to post-6-month BBFS exercise program (Table 2). However, when the data were sub-categorized into adherence groups, only those who attended at least 80% of the recommended BBFS visits significantly improved 6 MWD (Table 1; ≥80% Adherence, +37 ± 58 m; < 80% Adherence, −1 ± 105 m; between-group difference, *p* = 0.046). A similar pattern of change was evident after excluding those who reported a change in dopamine-related medications during the 6-month intervention period (Table 3). Our sub-analysis also showed that only those who have had a neurological diagnosis for < 6 years had a positive change in 6 MWD (Figure 2A; Time of diagnosis < 6 years, +39 ± 76 m; Time of diagnosis ≥6 years, −36 ± 123 m; between-group difference, *p* = 0.029). In individuals with a neurological diagnosis for 6 years or greater, only those who adhered to 80% of the recommended BBFS visits showed 6 MWD improvement (Figure 2B; ≥80% Adherence, +42 ± 64 m); < 80% Adherence, −67 ± 105 m).

### 3.2. Other objective measures of functional capacity and fall risk

In all participants, there were significant improvements in functional capacity outcomes including 30 s STS, 30 s bicep curl, and hamstring and shoulder flexibility following the 6-month BBFS intervention (Table 2). There were no significant changes in postural stability or fall risk measures (Table 2). A similar pattern of functional capacity and fall risk results was found after excluding those who reported changes in dopamine-related medication during the intervention period (Table 3), with greater improvements (30 s STS, TUG, 30 s bicep curl test, and shoulder flexibility) found in those who attended 80% of the recommended BBFS visits. It should be noted that an additional functional capacity improvement reached statistical significance following the 6-month program (TUG right side, −1 [−1 to 0] s, *p* = 0.023), and only one flexibility measure (hamstring left side, +4 ± 12, *p* = 0.031) showed statistical significance (Table 3). There were no statistically significant pre- to post-6-month changes in these objective outcome measures of functional capacity and fall risk between categories of time since neurological diagnosis (≥6 years vs. < 6 years).

#### 3.2.1. Subjective measures of functional capacity and fall risk

Analysis of modified FROP-Com, NFOG-Q and FESI showed no significant differences in subjective measures of functional capacity and fall risk from pre- to post-6-month BBFS exercise program or between the two adherence groups who attended at least 80 or < 80% of the recommended BBFS visits (*p* > 0.05, Table 2). In addition, there were no statistically significant pre- to post-6-month changes in these subjective measures of functional capacity and fall risk between categories of time since neurological diagnosis (≥6 years vs. < 6 years).

#### 3.2.2. Health-related quality of life

BBFS exercise program only led to minimal improvements in health-related quality of life as measured using PDQ-39, SF-12 (PCS-12 and MCS-12 summary scores), EQ-5D-3L and exercise self-efficacy questionnaires (Table 2). In all participants, we found no significant differences in health-related quality of life from pre- to post-6-month BBFS exercise program or between the two adherence groups (*p* > 0.05, Table 2).

## 4. Discussion

To the best of our knowledge, this is the first study to evaluate an AEP-led inter-professional healthcare service (BBFS), specializing in clients with ND. Although we found no statistically significant change in 6 MWD from pre- to post-6-month intervention (41), our sub-analysis showed a significant improvement in 6 MWD only in members who had at least 80% adherence to the BBFS recommended visits (≥80% Adherence, +37 ± 58 m; < 80% Adherence, −1 ± 105 m), even after excluding those who had a change in dopamine-related medications during the BBFS intervention period. However, this finding should still be interpreted with caution given the uneven number of participants between groups.

Nevertheless, this could be deemed as an important finding because a change in dopamine-related medications during the intervention period, which were taken by the majority of BBFS members included, may either have a detrimental or beneficial effect on the outcome measures of the present study, depending on the specific dosage changes. For example, those with an increase in dopamine-related medication during the program period could have had an additional outcome benefit beyond the impact of the BBFS exercise program. In this regard, our results suggest that a more regular adherence to the BBFS exercise program may improve 6 MWD beyond the effects of dopamine-related medications. However, a prospective randomized controlled trial is warranted to confirm this finding.

It is also noteworthy that only those in the early stage of their ND (time since diagnosis < 6 years) had a favorable change in 6 MWD after the 6-month BBFS program. Interestingly, when only looking at the members who are at the later stage of their disease (time since diagnosis ≥6 years), those who adhered to at least 80% of the BBFS program appeared to still benefit, showing a significant 6 MWD improvement. Collectively, our findings emphasize the importance of regular participation in the BBFS program, regardless of disease stage, in the improvement of FC, and plausible disease progression in people with ND. This should however be interpreted with caution due to the lack of specificity of the disease stage assessment used in this study. In the present study, the disease stage was only inferred from the self-reported time since diagnosis' of the clients” ND relative to the traditionally accepted methods such as staging using the Hoehn and Yahr scale (42). Nevertheless, this is an exciting finding which suggest that it is never too late to refer clients to a clinic such as the BBFS, to preserve or improve independent living in this population.

Moreover, although we found no statistically significant change in 6 MWD from pre- to post-intervention, a clinically meaningful improvement may be evident (Pre- vs. Post-6-month 6MWD change: +15 ± 90). Indeed, a systematic review conducted by Bohannon et al. (41) reported that a 6 MWD improvement of at least 14 m is clinically meaningful in adults with pathology. This clinically meaningful change in 6 MWD [>14 m; (41)] following the 6-month BBFS intervention was accompanied by significant changes in other secondary objective functional capacity and fall risk measures (30 s STS, TUG, 30 s bicep curl). These results are consistent with previous findings showing functional capacity improvement following 3 to 6 months of cardiorespiratory training (43–46), resistance training (47), alternative training such as boxing (34) and dancing (35), or a combination of these training modes (48), which were all delivered as components of the BBFS multimodal exercise program. Exercise training has long been established to promote neuroplasticity and attenuate the risk and progression of neurodegenerative diseases (49, 50), which is likely mediated by the brain-derived neurotrophic factor (BDNF) (49, 51–55). Specifically in people with Parkinson's disease, which represents the majority (85%) of participants in the present study, BDNF and exercise have been shown to stimulate growth and survival of dopaminergic neurons in the substantia nigra pars compacta (56). This could explain the clinical improvements in 6 MWD and other objective functional capacity and fall risk measures found in the present study, plausibly reflecting the attenuation of motor and non-motor symptoms which usually arise from the deterioration of dopaminergic neurons in the substantia nigra pars compacta in people with Parkinson's disease (57). Moreover, the variety of exercise modes delivered as part of the BBFS service presents the need to learn new movements or motor patterns which may have contributed to an increase or maintenance of dopaminergic levels. Indeed, it has been demonstrated that the acquisition of new motor patterns compared to the performance of the same repeated movement pattern may result in better dopamine-induced activation of the striatal region (58) to facilitate smooth and fluid voluntary movement that may enable performance of safe and independent activities of daily living.

However, our objective findings are not reflected by our subjective outcomes of functional capacity and fall risk determined from standardized questionnaires including the Modified Frop-Com, NFOG-Q, and FESI, which were only administered to BBFS members diagnosed with Parkinson's Disease. This is not at all surprising given that a recent study showed that the NFOG-Q questionnaire is insufficiently reliable or responsive to detect small effect sizes (59), suggesting that the use of subjective questionnaires to assess a change in FC and fall risk may be unsuitable as outcomes in clinical trials. It should also be noted that our baseline data derived from these subjective questionnaires already indicate relatively normal functional capacity and low fall risk, limiting the room for a significant improvement to occur. For example, the average baseline total score for the modified Frop-Com found in the present study was 3 ± 5, indicating a low risk of fall (16) even before the initiation of the BBFS program. Thus, the absence of a significant change in these subjective outcome measures from pre- to -post-intervention may instead indicate the ability of the BBFS program to maintain functional capacity and low falls risk in this population. This is an important finding because an older adult cohort in combination with a neurological disorder, is expected to have a certain degree of functional decline and thus risk of falls over 6 months, which we found to be preserved by the BBFS program.

The PDQ-39 is a commonly used self-reported questionnaire, which assesses Parkinson's disease specific health related quality of life across eight dimensions (31, 32). In this study, we did not find any significant change in QOL in clients with PD after 6-months of the BBFS intervention. This could be due to the inherent challenges in the interpretation and validity of PDQ-39 summary index scores (60). It is known that people with PD are sensitive to a wide range of non-motor symptoms, and traditional questionnaires (such as PDQ-39) may not be accurate to capture these symptoms in PD (61, 62). Therefore, other questionnaires, such as the Neuro-QOL, (63) may be more appropriate in assessing the effects of exercise on these non-motor health-related QOL parameters in patients with PD. Whether longer-term adherence to exercise (>6 months) improve health related QOL in this population of ND warrants further investigation.

Another noteworthy finding of this evaluation is that only 42 of the BBFS members were able to adhere to 80% of the recommended center-based BBFS visits after 6-months. Consistent with previous reports in adults with and without physical disabilities (64), one of the most often reported barrier to adherence to the center based BBFS program is lack of transportation. Indeed, anecdotally, BBFS members appeared to be more dependent on partners or carers for transport. For example, a BBFS member reported not being able to adhere to the program when his wife suffered from a major illness and a fall, despite being highly motivated to participate in the BBFS program. Given that the present study and others (65, 66) reveal a more favorable clinical outcome with regular adherence to an exercise intervention, an expansion of the BBFS program to remote delivery *via* telehealth, self-managed home-based program, or a hybrid of center-based and home-based sessions may be warranted. The socioeconomic status of the patients can also impact their participation, compliance to exercise and associated health outcome (67), which wasn't specifically analyzed in this study and should be assessed in future longitudinal studies.

### 4.1. Limitations

The main limitation of this study is its retrospective design. The study relied on data which were already collected by the AEPs of the BBFS clinic. A future study with a more rigorous research design, such as a prospective parallel designed randomized trial, may better validate the outcomes of the present study. The lack of a control group is also considered a major limitation of this study as it limited our ability to determine if the identified change is beyond the variability and technical measurement error of our desired outcome measures. Our results should be taken with caution until larger and more rigorous research trials are conducted. Nevertheless, a significant relative improvement in FC, especially in participants with >80% adherence to the BBFS program, provided important and novel insights into the efficacy of individualized evidence-based multimodal exercise prescription in improving FC, muscular strength, and flexibility in patients with ND. It should also be noted that most of the included participants of this study are diagnosed with PD, which makes it difficult to generalize our results to people with other ND. Nevertheless, the outcomes of this study reflect the motor and non-motor symptom progression that are common across a variety of ND. A more robust FC and fall risk measure are also warranted, however, the outcome assessments used in the present study are typically performed in most healthcare clinics nationally and internationally, increasing the translational aspect of this study.

## 5. Conclusion

The BBFS is one of the first AEP-led inter-professional health care services in Adelaide, South Australia to provide individualized evidence-based multimodal exercise prescription and social support for people with ND. In this study, we found that adhering to the exercise program at the BBFS center-based clinic for 6 months could significantly improve FC in individuals with ND as indicated by a clinically meaningful improvement in 6 MWD, which was greater in members who attended at least 80% of the recommended visits. 6 months of the BBFS intervention also resulted in significant improvements in muscular strength (30 s STS, bicep curls for both arms) and flexibility (hamstring both sides and right shoulder) in all participants. The improvements in functional capacity was also generally greater for individuals in the early stage of their ND (time since diagnosis < 6 years). However, these favorable changes in functional capacity did not translate into overall QOL improvement in this cohort. Future longitudinal studies with larger sample sizes and more sensitive instruments for capturing QOL measures and perceived functional capacity and falls risk are warranted to investigate whether improvements in these clinical outcomes can be retained over a longer period and translated to improved QOL in individuals with ND.

## Data availability statement

The original contributions presented in the study are included in the article/supplementary material, further inquiries can be directed to the corresponding author.

## Ethics statement

The studies involving human participants were reviewed and approved by Human Research Ethics Committee at Flinders University. The patients/participants provided their written informed consent to participate in this study.

## Author contributions

JR wrote the manuscript, analyzed, and interpreted the data. RC also contributed to the write-up, analysis, and interpretation of sections pertaining to the secondary health-related QOL measures of the manuscript. JR, RC, LD, KS, JK, TH, and ON reviewed and edited and also provided input on the final draft of the manuscript. TH, ON, and their BBFS team collected the data. JR is the guarantor of this study. All authors contributed to the article and approved the submitted version.
